# Imaging-based development and validation of artificial intelligence models for lung adenocarcinoma precursor lesions and early lung adenocarcinoma presenting as pulmonary nodules

**DOI:** 10.3389/frai.2026.1889248

**Published:** 2026-09-15

**Authors:** Junbao Zhang, Yanyi Hou, Yikang Yang, Ziyan Zhang, Yi Gao, Ping Xu

**Affiliations:** 1 Department of Pulmonary and Critical Care Medicine, Peking University Shenzhen Hospital, Shenzhen, China; 2 Department of Pulmonary and Critical Care Medicine, Huashan Hospital, Fudan University, Shanghai, China; 3 Shenzhen University Medical School, Shenzhen, China; 4 Department of Radiotherapy Oncology, Sun Yat-sen Memorial Hospital, Sun Yat-sen University, Guangzhou, China; 5 Department of Radiation Oncology, Sichuan Cancer Hospital and Institute, University of Electronic Science and Technology of China, Chengdu, China

**Keywords:** DINOv3, invasiveness prediction, lung adenocarcinoma, multimodal learning, pulmonary nodules

## Abstract

**Background:**

Accurate preoperative assessment of pulmonary nodule invasiveness remains challenging. We developed an internally validated multimodal framework integrating CT representations from a frozen vision foundation model with clinical variables.

**Methods:**

This retrospective single-centre study included 1,179 pathologically confirmed pulmonary nodules: 247 glandular precursor lesions comprising atypical adenomatous hyperplasia and adenocarcinoma *in situ*, and 932 invasive lesions comprising minimally invasive and invasive adenocarcinoma. CT volumes were resampled to 0.5-mm isotropic resolution and cropped into 64 × 64 × 64-voxel patches. Slice-level representations were extracted using a pretrained, frozen DINOv3 backbone and aggregated by a trainable Attention Probe. Encoded clinical variables and imaging representations were integrated through self-attention and bidirectional cross-attention, followed by neural classification and regression tree classification. Internal validation used a five-fold rotating train–validation–test procedure, with each fold serving once as the held-out test fold.

**Results:**

The held-out test-fold AUCs were 0.848, 0.864, 0.867, 0.882, and 0.822, yielding a mean AUC of 0.8566. Pooled out-of-fold predictions produced an AUC of 0.847, accuracy of 0.809, sensitivity of 0.806, specificity of 0.822, and F1 score of 0.873. DINOv3 and NCART achieved the highest point-estimate AUCs among the evaluated feature extractors and classifiers, respectively, although most pairwise differences were not statistically significant. Intermediate fusion significantly outperformed the Gould score and the clinical-data-only model, but not the imaging-only or late-fusion models. In the prespecified secondary analysis, the model achieved an AUC of 0.780 for distinguishing adenocarcinoma *in situ* from atypical adenomatous hyperplasia.

**Conclusion:**

The proposed framework achieved internally validated discrimination of pulmonary nodule invasiveness. External multicentre and prospective validation is required before clinical implementation.

## 1 Introduction

Lung cancer remains the leading cause of cancer-related mortality globally. In 2022, there were 2.5 million new cases and 1.8 million deaths (Bray et al., 2024). It is estimated that by 2050, the annual number of lung cancer cases will reach 3.8 million (Leiter et al., 2023). Recognizing early-stage lung cancer is crucial, as the 5-year survival rate for Stage IA is 80% higher than for Stage IV (Jeon et al., 2023; Wang et al., 2023). Early lung cancer typically presents as pulmonary nodules. Due to an aging population and increased chest CT screenings post-COVID, detection rates have surged, reaching 50% among health-checkup populations in China in 2023. Remarkably, up to 96% of these nodules are benign (Silva et al., 2021). Lung adenocarcinoma has recently surpassed squamous cell carcinoma as the most common pathological type (WHO Classification of Tumours Editorial Board, 2021). According to the 2021 WHO classification, it ranges from AAH, AIS to MIA and IAC (Tsao et al., 2022). Different degrees of infiltration dictate distinct follow-up protocols, interventions, and prognoses. Therefore, detecting pathological infiltration necessitates early surgical treatment (Miller et al., 2022; Liu et al., 2022).

While traditional imaging relies on subjective interpretation, Radiomics allows for quantitative data extraction. In 2012, Lambin and others proposed Radiomics, that is concepts such as imaging genomics. The team believed that more quantitative information could be obtained from imaging methods other than CT (Lambin et al., 2012). It is a major innovation in contemporary medicine. Recently, AI technologies utilizing Convolutional Neural Networks and deep learning have enabled fully automated segmentation and quantitative analysis of 3D nodule structures (Fang et al., 2022). Now, a self-supervised foundational model, DINOv3, has demonstrated robust feature learning from large-scale, unlabeled image data. However, clinical metadata is often excluded from these models.

This study aimed to develop and internally validate a multimodal framework for predicting pulmonary nodule invasiveness by integrating CT representations extracted using a pretrained and frozen DINOv3 backbone with clinical variables through an attention-based fusion strategy. Postoperative pathological diagnosis was used as the reference standard for distinguishing invasive lesions comprising MIA and IAC from glandular precursor lesions comprising AAH and AIS.

## 2 Methods

### 2.1 Data source

In this study, preoperative CT scans from 1,264 patients were collected at Peking University Shenzhen Hospital. Patients’ clinical information was retrieved from the hospital’s electronic medical record system, including sex, age, history of malignancy, smoking history, and pathological findings. For patients with multiple pulmonary nodules, each nodule was considered an independent primary lesion.

### 2.2 Definition of the primary endpoint and secondary subgroup analysis

The primary study endpoint was the binary classification of pulmonary nodules according to pathological invasiveness. Based on postoperative pathological findings, MIA and IAC were categorized as invasive lesions and defined as the positive class, whereas AAH and AIS were categorized as glandular precursor lesions and defined as the negative class. The primary model was therefore developed to distinguish invasive lesions from glandular precursor lesions.

A prespecified secondary subgroup analysis was conducted among all pathologically confirmed AAH and AIS lesions. In this analysis, AIS was defined as the positive class and AAH as the negative class. This secondary analysis was separate from the primary endpoint and did not depend on the predictions generated by the primary model. AAH, AIS, MIA, and IAC were not modeled simultaneously as four independent outcome classes.

### 2.3 Inclusion and exclusion criteria for CT images

To ensure data quality and diagnostic accuracy, the study population was restricted to patients with nodules confirmed to be benign or malignant. The diagnosis was established based on pathological examination, including biopsy and surgical resection. In addition, exclusion criteria were defined to remove cases that could compromise the reliability of the analysis. The exclusion criteria were as follows:

(a) CT scans with a slice thickness greater than 2.0 mm were excluded not only due to reduced image quality, but also because small pulmonary nodules may be partially visualized or missed entirely, which could adversely affect image analysis.
(b) Cases were excluded if two or more pulmonary nodules were present and the distance between any two nodules was less than 42 mm within the axial plane or less than 80 mm along the craniocaudal direction, in order to reduce the risk of incorrectly matching pathological findings to the corresponding nodules on CT.
(c) Cases with incomplete clinical diagnostic information were excluded.

Figure 1 illustrates the patient recruitment workflow.

**Figure 1:**
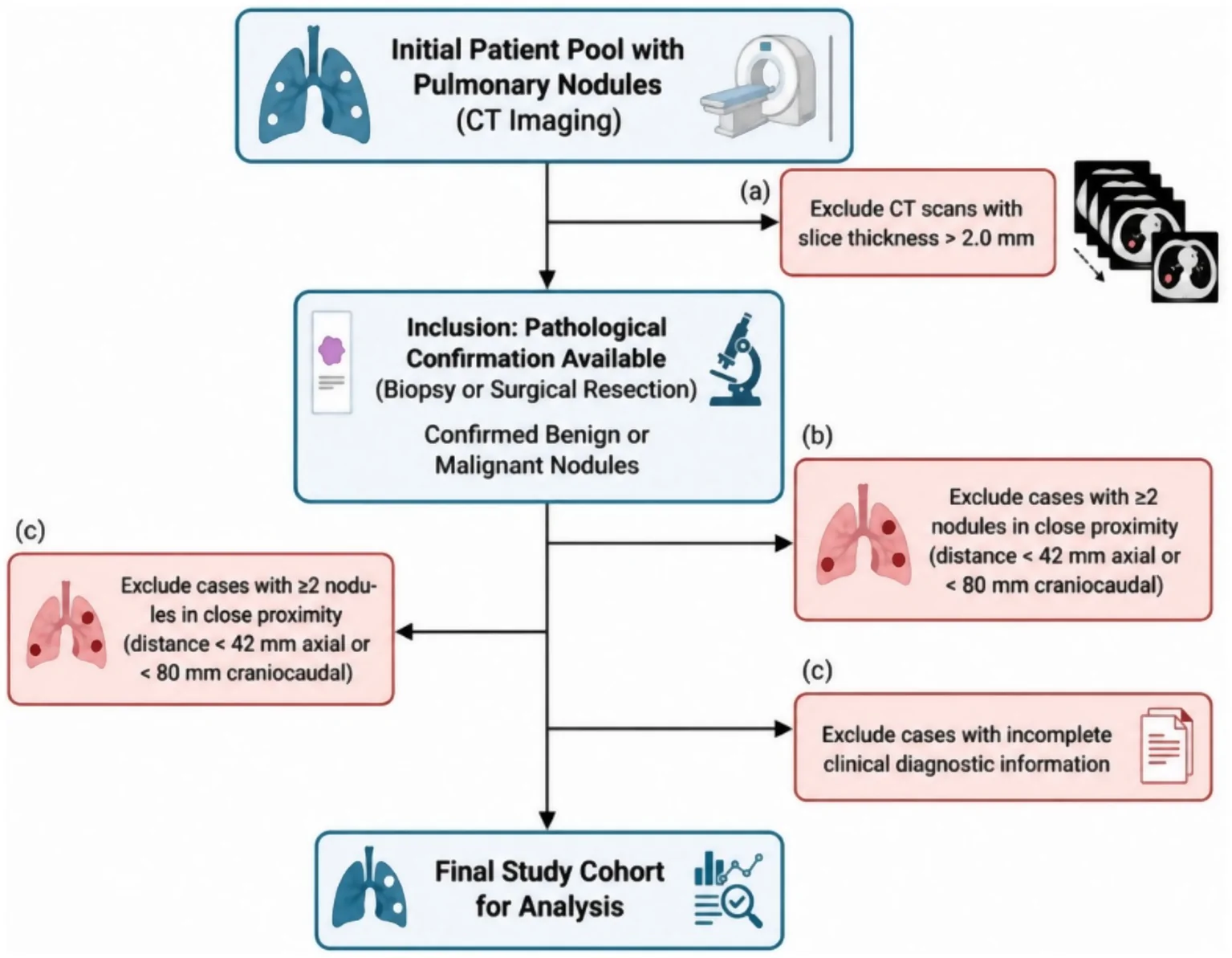
Flow diagram of patient selection and study cohort construction. Patients with pulmonary nodules identified on CT imaging and with pathological confirmation were screened for eligibility. CT scans with a slice thickness greater than 2.0 mm, cases with multiple nodules in proximity, and cases with incomplete clinical diagnostic information were excluded.

### 2.4 Nodule localization and ROI generation

All CT volumes were resampled by interpolation to an isotropic voxel spacing of 0.5 × 0.5 × 0.5 mm before ROI extraction.

Target pulmonary nodules were manually identified by two radiologists experienced in thoracic CT interpretation. No automated nodule-detection algorithm or semi-automatic segmentation software was used. The radiologists independently reviewed the CT images and identified the target lesion by cross-referencing its anatomical location with the corresponding pathological record. For patients with multiple pulmonary nodules, the target lesion was matched according to the involved pulmonary lobe, anatomical position, nodule size, and radiological characteristics. Any disagreement regarding lesion identity or location was resolved by consensus.

After confirmation of the target lesion, a researcher trained in thoracic CT image interpretation manually placed an approximate centre point within the nodule using open-source medical imaging software (3D Slicer, version 5.6.1). The reference point was positioned to reasonably represent the centre of the lesion and was not derived from a pixel-level or voxel-level segmentation mask. The locations of the reference points and the corresponding extracted image patches were subsequently reviewed by the radiologists.

A fixed 64 × 64 × 64 voxel patch was extracted around each confirmed reference point, corresponding to an approximate physical field of view of 32 × 32 × 32 mm. When the standard cropping window extended beyond the CT image boundary, the window was shifted toward the opposite direction to ensure that the extracted patch contained only valid CT voxels. In these cases, the target nodule was not necessarily located at the exact geometric centre of the patch. The extracted patch contained the target nodule together with a limited amount of surrounding pulmonary tissue and was used as the imaging input for model development.

### 2.5 Model training

This study proposes a novel deep learning network architecture designed to extract and fuse features from CT images and clinical data, aiming to achieve optimal lung nodule classification. The architecture of the model is shown in Figure 2 and consists of three modules:

1. A DINOv3-based encoder for CT images and an advanced feature embedding module for clinical data.
2. A feature fusion module that integrates intra- and inter-data relationships through self-attention and cross-attention mechanisms.
3. A machine learning classifier that models the fused features and outputs the final classification results.

**Figure 2:**
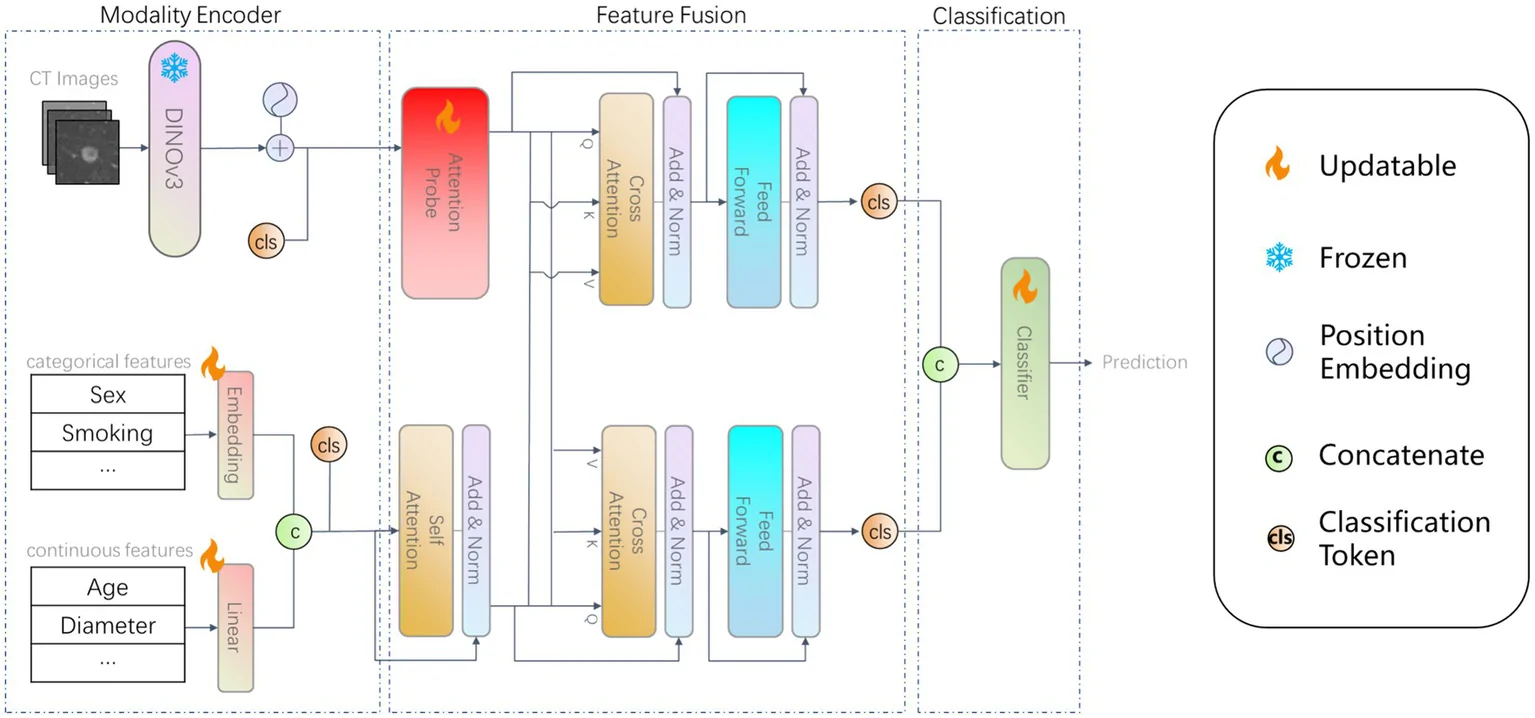
Architecture of the proposed multimodal framework with a frozen DINOv3 backbone. The snowflake symbol indicates the frozen DINOv3 backbone, whereas the flame symbol indicates trainable downstream modules, including the Attention Probe, clinical feature encoders, multimodal fusion components, and the classifier. The CT representations extracted by DINOv3 and the encoded categorical and continuous clinical features are integrated through self-attention and bidirectional cross-attention before final classification.

#### 2.5.1 Foundation models

Vision foundation models refer to general-purpose models that are pre-trained at large scale on massive image, video, and multimodal datasets and can be adapted to a variety of downstream vision tasks with only limited fine-tuning. Their backbone networks produce stable and generalizable visual features, fully demonstrating their powerful representation learning capability. Masked Autoencoders (MAE) are a prominent example. By randomly masking portions of an image and reconstructing the missing content using only the unmasked information, MAE learns image representations in a self-supervised manner (He et al., 2021). After pre-training and fine-tuning, MAE achieves excellent performance on classification tasks. However, because significant differences exist between natural images and medical images, further adaptation and fine-tuning are usually required to achieve optimal performance in clinical applications.

The DINO series represents a particularly promising class of self-supervised foundation models. DINOv2 has demonstrated that Transformers pre-trained on natural images can produce stable features, enabling their frozen backbone networks to transfer effectively to a wide range of downstream tasks (Oquab et al., 2023). The latest version, DINOv3, stands out even further. It is a self-supervised Vision Transformer that learns representations from large-scale unlabeled natural image data without relying on explicit human annotations (Siméoni et al., 2025). This training strategy helps the model develop a more comprehensive understanding of diverse visual scenes, including image texture, shape, and spatial relationships. As an outstanding foundation model, DINOv3 can provide stable performance across multiple tasks with only a single forward pass, thereby significantly reducing computational resources and cost. In addition, its Gram Anchoring mechanism enables the generation of higher-quality feature maps.

#### 2.5.2 Feature encoding

DINOv3, pretrained on the LVD-1689 M dataset, was used to encode the CT image inputs, whereas separate trainable encoding modules were used for the clinical variables. These modules generated imaging and clinical representations for subsequent multimodal fusion.

##### 2.5.2.1 CT image encoding

The image encoder was built upon a pretrained and frozen DINOv3 backbone. All parameters of the DINOv3 backbone, including those of the Vision Transformer encoder, remained fixed throughout model training and were not updated during optimization.

Each CT volume was divided into 64 axial slices, which were sequentially passed through the frozen DINOv3 backbone to extract the corresponding high-level image representations. The DINOv3 classification token obtained from each slice was retained, and the classification tokens from all 64 slices were concatenated to form a two-dimensional sequential representation. This representation was subsequently processed by the trainable Attention Probe module.

Because the self-attention mechanism within the Attention Probe could not inherently capture the relative positions of the individual axial slices, positional encoding was added to the DINOv3 classification token of each slice before attention-based processing.

##### 2.5.2.2 Categorical clinical feature encoding

Categorical clinical features, including binary 0–1 variables, were transformed into d -dimensional trainable embedding vectors using a strategy similar to those commonly adopted in natural language processing. Each categorical feature was represented by an independent embedding function.

For the i-th categorical feature $x_{i}$ , its embedding function was denoted by $e_{\phi_{i}}(\cdot )$ . If the feature contained $s_{i}$ possible observed categories, a total of $s_{i}+1$ embedding vectors were defined, with the additional embedding used to represent missing values.

Each categorical embedding was constructed by concatenating two components. The first component, $c_{\phi_{i}}$ , was a feature-specific identifier used to distinguish different feature fields, such as sex and smoking history. This identifier had dimensionality l and was shared by all categories within the same feature. The second component, $w_{\phi_{i}j}$ , was a category-specific embedding vector used to distinguish individual category values within the same feature. For example, for the feature sex, separate category-specific embeddings were assigned to the categories male and female. This component had dimensionality d−l .

For the i-th feature with the observed value $x_{i}=j$ , where j represented one of the observed categories or the missing-value category, its embedding was defined as follows:


$$e_{\phi_{i}}(x_{i})=[c_{\phi_{i}};w_{\phi_{i}j}],$$ (1)

where


$$c_{\phi_{i}}\in {\mathbb{R}}^{l},\;\;\;\;w_{\phi_{i}j}\in {\mathbb{R}}^{d-l}.$$ (2)

Here, the semicolon denotes vector concatenation.

This embedding strategy was proposed by Huang et al. (2020) and enabled the generation of distinct dense representations for individual categorical clinical features. Unlike textual sequences, tabular data do not possess an inherent positional order among feature columns; therefore, conventional positional encoding was not considered appropriate for distinguishing tabular variables. Instead, the feature-specific identifier $c_{\phi_{i}}$ was introduced to explicitly encode the identity of each feature.

Compared with one-hot encoding, which produces sparse numerical representations, the trainable embedding layer assigned each categorical clinical variable a dense representation that could be jointly optimized during model training. To facilitate subsequent multimodal feature fusion, the dimensionality of each categorical embedding was set to match the output dimensionality of the Attention Probe module.

##### 2.5.2.3 Continuous clinical feature encoding

Continuous clinical features were likewise projected into d -dimensional embedding vectors. For each continuous feature, its empirical distribution was estimated using only the training data. The feature space was then partitioned into T non-overlapping intervals, denoted by $B_{1},B_{2},\ldots ,B_{T}$ , which were referred to as bins.

For the i-th continuous feature, the t-th interval was defined as follows:


$$B_{i,t}=[b_{i,t-1},b_{i,t}],\;\;\;\;t=1,\ldots ,T.$$

The boundaries of these intervals were determined using quantiles calculated from the training data. For the i-th continuous feature $x_{i}$ , the t-th quantile boundary was defined as follows:


$$b_{i,t}=Q_{t/T}(\{x_{i}^{\left(j\right)}:j\in J_{\text{train}}\}),\;\;\;\;t=0,\ldots ,T,$$

where $Q_{t/T}$ denotes the quantile function at probability t/T , and $J_{\text{train}}$ denotes the index set of the training samples.

Piecewise-linear encoding was then adopted to generate informative representations of the continuous clinical variables. For an observed value $x_{i}$ , the encoding was defined as follows:


$${\mathrm{PLE}}_{i}(x_{i})=[e_{i,1}(x_{i}),,\ldots ,,e_{i,T}(x_{i})]\in {\mathbb{R}}^{T}$$

For the t-th bin, the corresponding encoded component was calculated as follows:


$$e_{i,t}(x_{i})=\{\begin{array}{ll} 0, & x_{i}<b_{i,t-1},\\ 1, & x_{i}\geq b_{i,t},\\ \frac{x_{i}-b_{i,t-1}}{b_{i,t}-b_{i,t-1}}, & \text{otherwise}. \end{array}$$

This piecewise-linear encoding method was introduced by Gorishniy et al. (2022) for representing numerical tabular data in deep learning models.

For attention-based architectures, it was also necessary to incorporate information identifying the original clinical feature associated with each numerical representation. Therefore, a feature-specific linear layer was applied after the piecewise-linear encoding transformation. The parameters of this linear layer were not shared across different continuous features.

Accordingly, the final encoding of the i-th continuous feature was expressed as follows:


$$f_{i}(x_{i})=b_{i}+\sum_{t=1}^{T}e_{i,t}(x_{i})w_{i,t}={\text{Linear}}_{i}\;({\mathrm{PLE}}_{i}(x_{i})),$$

where $w_{i,t}$ and $b_{i}$ denote the feature-specific weight vectors and bias term, respectively. The output dimensionality of $f_{i}(x_{i})$ was set to d , thereby matching the dimensionality of the categorical embeddings and the representations generated by the Attention Probe module.

#### 2.5.3 Feature fusion

In this study, feature information from different modules was processed through a series of specialized layers. The self-attention layer was used to generate contextual representations among embeddings of different clinical variables. The attention probe module was employed to process the two-dimensional sequence embeddings generated by DINOv3 and to capture feature information across individual slices. The cross-attention mechanism was used to explore the dependencies between imaging and clinical information, whereas the feed-forward network was applied to model the nonlinear relationships between radiological and clinical features.

Self-attention mechanism — In this study, the self-attention layer proposed by Vaswani et al. (2017) was adopted to enhance information fusion and transmission within each modality, thereby generating more informative feature representations. The attention probe module consisted of a multi-head self-attention layer and two feed-forward layers, with layer normalization and residual connections applied between layers. The attention probe module was designed to capture the dependencies among the two-dimensional slice-level classification tokens output by DINOv3. In contrast, the self-attention layer applied to categorical and numerical clinical features was used to model the dependencies among all clinical variables. The self-attention mechanism can be formulated as follows:


$$\text{Attention}(Q,K,V)=\text{softmax}(\frac{QK^{T}}{\sqrt{d_{k}}})V$$

Each self-attention layer was used to capture complex intra-modal relationships. Its output integrated information from all input vectors, thereby producing richer semantic representations.

Cross-attention mechanism — The cross-attention mechanism was used to enable information interaction between two different modalities and to fuse contextual embeddings from both modalities. This mechanism is similar to self-attention, except that the query vector (Q) is derived from one modality, whereas the key (K) and value (V) vectors are derived from the other modality. It can be expressed as follows:


$$\text{Attention}(Q_{\mathrm{img}},K_{\text{clin}},V_{\text{clin}})=\text{softmax}(\frac{Q_{\mathrm{img}}{K_{\text{clin}}}^{T}}{\sqrt{d_{k}}})V_{\text{clin}}$$

$$\text{Attention}(Q_{\text{clin}},K_{\mathrm{img}},V_{\mathrm{img}})=\text{softmax}(\frac{Q_{\text{clin}}{K_{\mathrm{img}}}^{T}}{\sqrt{d_{k}}})V_{\mathrm{img}}$$

where img and clin denote CT imaging features and clinical tabular information, respectively. The above strategy enhances the interaction between the two modalities. The fusion strategy was inspired by Amador et al. (2025) and integrates the imaging representations processed by the trainable Attention Probe module with the representations of the clinical tabular data. Specifically, the image tokens obtained from Equation 1 can be interpreted as local image features augmented with the most relevant clinical information, enabling each image feature to incorporate clinically meaningful evidence for diagnosis. Conversely, the tabular tokens obtained from Equation 2 represent clinical information enriched with the most relevant imaging features, allowing clinical tabular data to move beyond isolated static values and to be accompanied by multimodal explanations supported by imaging evidence.

### 2.6 Experiments

The proposed framework was implemented using PyTorch. Comparative experiments were conducted to evaluate different CT feature extractors, classification algorithms, modality configurations, and multimodal fusion strategies. All candidate configurations were assessed under the predefined model-development and evaluation framework. Detailed implementation settings, model evaluation procedures, experimental objectives, model-comparison settings, feature-visualization methods, and statistical analyses are described in Sections 2.6.1–2.6.6.

#### 2.6.1 Implementation details

The proposed network was implemented using the PyTorch framework and trained on a workstation equipped with eight NVIDIA RTX A6000 GPUs, each with 48 GB of memory. The DINOv3 backbone remained frozen throughout training. The trainable downstream components, including the Attention Probe, clinical-feature encoding modules, multimodal fusion modules, and NCART classification module, were jointly optimized using AdamW with an initial learning rate of 1 × 10^−5^ and a cosine-annealing learning-rate schedule. The batch size was set to 32, the number of worker threads to 4, and the maximum number of training epochs to 400. For the feature-extractor comparison, the same optimization settings were applied to all trainable parameters, although the initialization strategies differed as described in Section 2.6.3.

#### 2.6.2 Model evaluation

All 1,179 pulmonary nodules were evaluated using a five-fold cross-validation framework. The dataset was partitioned into five mutually exclusive folds. Partitioning was performed at the patient level; therefore, all nodules from the same patient were assigned to the same fold, and no patient appeared in more than one fold. In each iteration, three folds, representing approximately 60% of the cohort, were used for model training; one fold, representing approximately 20%, was used for validation and selection of the best-performing model checkpoint; and the remaining fold, representing approximately 20%, was retained as the held-out test fold. The held-out test fold was not used during model training, optimization, or model selection.

The procedure was repeated for five iterations so that each fold served once as the held-out test fold. Model performance in each iteration was evaluated on the corresponding held-out test fold. The area under the receiver operating characteristic curve, accuracy, precision, sensitivity, specificity, and F1 score were used as performance metrics. The fold-specific AUCs and their arithmetic mean were reported as the primary internal-validation results.

In addition to reporting the fold-specific AUCs and their arithmetic mean, the predictions from the five held-out test folds were pooled to generate one out-of-fold prediction for each of the 1,179 nodules. These pooled out-of-fold predictions were used to calculate the overall performance metrics and their 95% confidence intervals and to conduct comparisons between candidate models.

#### 2.6.3 Experimental objectives and settings

To compare different CT feature-extraction strategies, five architectures were evaluated: VGG-16, ResNet-50, DenseNet-121, ViT, and DINOv3. VGG-16 (Simonyan and Zisserman, 2014), ResNet-50 (He et al., 2015), DenseNet-121 (Dey et al., 2018), and ViT were randomly initialized and trained from scratch on the pulmonary nodule dataset. DINOv3 was initialized using its officially released self-supervised pretrained weights from the LVD-1689 M dataset, and the entire DINOv3 backbone remained frozen throughout training. Its extracted CT representations were subsequently processed by the trainable Attention Probe, which served as a lightweight CT-domain adapter.

Apart from differences in parameter initialization and pretraining status, all feature extractors were connected to the same downstream fully connected classification head and evaluated using the same data partitions, loss function, optimizer, learning-rate schedule, batch size, training duration, data-augmentation procedure, and evaluation protocol. Because the initialization strategies differed, this experiment was regarded as a pragmatic comparison of feature-extraction strategies under the implemented training conditions rather than a strictly controlled architectural ablation.

To further investigate the contributions of different data modalities and fusion strategies, we compared the proposed intermediate-fusion model with independently trained imaging-only and clinical-data-only models, as well as a multimodal late-fusion model. The architectures of the two unimodal models are shown in Supplementary Figure S1. In the imaging-only model, the pretrained and frozen DINOv3 backbone was used to extract CT representations, which were subsequently processed by a trainable Attention Probe incorporating positional encoding and self-attention before classification. In the clinical-data-only model, categorical and continuous clinical variables were encoded separately, concatenated to form a unified clinical representation, and then passed directly to the classifier. The architecture of the late-fusion baseline is shown in Supplementary Figure S2. In this model, the imaging and clinical representations were processed independently and concatenated immediately before the final classifier. In addition, the widely used prediction model developed by Mayo Clinic researchers and adopted in the ACCP guidelines, CSP 027 (Gould et al., 2007), was included as one of the baseline methods for comparison.

#### 2.6.4 Comparative model-selection procedure

Candidate configurations were systematically compared for three components of the proposed framework: CT feature extraction, classification, and multimodal fusion. The evaluated feature extractors included VGG-16, ResNet-50, DenseNet-121, ViT, and DINOv3; the evaluated classifiers included a fully connected neural classifier, logistic regression, support vector machine, random forest, XGBoost, and NCART; and the evaluated modality and fusion configurations included the Gould score, clinical-data-only, imaging-only, late-fusion, and intermediate-fusion models.

For each model component, the configuration with the highest point-estimate AUC under the predefined evaluation framework was selected for inclusion in the final pipeline. Statistical comparisons of correlated AUCs were performed separately using the DeLong test, as described in Section 2.6.6. Detailed performance results are reported in Table 1 and Supplementary Tables S1–S3.

**Table 1:** Performance comparison of different image feature extraction strategies under identical training and evaluation settings.

Model	AUC(95% CI)	ACC(95% CI)	PREC(95% CI)	SEN(95% CI)	SPEC(95% CI)	F1(95% CI)
DenseNet-121	0.719 (0.649–0.788)	0.657 (0.594–0.714)	0.915 (0.854–0.952)	0.628 (0.557–0.694)	0.771 (0.635–0.867)	0.744 (0.691–0.795)
ResNet-50	0.718 (0.645–0.791)	0.617 (0.556–0.678)	0.939 (0.880–0.970)	0.565 (0.495–0.634)	0.844 (0.712–0.923)	0.706 (0.644–0.760)
VGG-16	0.679 (0.594–0.764)	0.530 (0.466–0.592)	0.926 (0.854–0.963)	0.455 (0.386–0.526)	0.844 (0.712–0.923)	0.611 (0.545–0.671)
ViT	0.715 (0.630–0.799)	0.699 (0.638–0.754)	0.902 (0.842–0.941)	0.694 (0.624–0.755)	0.720 (0.583–0.825)	0.784 (0.733–0.829)
DINOv3	0.729 (0.659–0.799)	0.648 (0.585–0.706)	0.920 (0.859–0.956)	0.612 (0.540–0.678)	0.792 (0.657–0.883)	0.735 (0.682–0.788)

VGG-16, ResNet-50, DenseNet-121, and ViT were randomly initialized and trained from scratch, whereas DINOv3 used frozen self-supervised pretrained weights from LVD-1689 M. Apart from initialization and pretraining status, all models used the same downstream classification head, training settings, data partitions, and evaluation protocol.

#### 2.6.5 Feature visualization

To qualitatively visualize the image representations extracted by DINOv3, principal component analysis was applied to the extracted feature embeddings. The first three principal components were mapped to the red, green, and blue channels, respectively, to generate pseudo-colour RGB feature visualizations. These visualizations were used to examine the spatial distribution of the learned image representations and were not used for model training, model selection, or quantitative performance evaluation.

#### 2.6.6 Statistical analysis

Clinical and conventional radiological characteristics were compared across the representative training, validation, and held-out test subsets using one-way analysis of variance for age and nodule diameter, the Kruskal–Wallis test for tumour markers, and the chi-square test or Fisher’s exact test for categorical variables, as appropriate. For comparisons according to pathological invasiveness, age was compared using Welch’s t-test, whereas nodule diameter and tumour-marker concentrations were compared using the Wilcoxon rank-sum test. Categorical variables were compared using the chi-square test or Fisher’s exact test, as appropriate, with Yates’ continuity correction applied to 2 × 2 chi-square tests. Continuous data are presented as mean ± standard deviation or median (interquartile range), as appropriate, and categorical data are presented as *n* (%).

Overall model-performance metrics and their 95% confidence intervals were calculated using pooled out-of-fold predictions from all five held-out test folds. Each of the 1,179 nodules therefore contributed one prediction generated when it belonged to the held-out test fold. Differences in discriminative performance between models were assessed using paired DeLong tests for correlated receiver operating characteristic curves based on these pooled out-of-fold predictions. Prespecified comparisons included DINOv3 versus each alternative feature extractor, NCART versus each alternative classifier, and the intermediate-fusion model versus each alternative modality or fusion strategy. Complete pairwise *p*-value matrices are provided in Supplementary Table S3.

Bootstrap resampling with 1,000 iterations was used to estimate 95% confidence intervals for the performance metrics. All statistical tests were two-sided, and *p* < 0.05 was considered statistically significant.

## 3 Results

### 3.1 Study population and clinical and radiological characteristics

A total of 1,179 pathologically confirmed pulmonary nodules were included, comprising 247 glandular precursor lesions, including AAH and AIS, and 932 invasive lesions, including MIA and IAC. According to the prespecified outcome definitions, the primary binary classification task was to distinguish invasive lesions from glandular precursor lesions. A prespecified secondary subgroup analysis was conducted among all pathologically confirmed AAH and AIS lesions to differentiate AIS from AAH.

The characteristics of the overall cohort and the data subsets in one representative iteration of the five-fold cross-validation procedure are summarized in Table 2. The training, validation, and held-out test subsets comprised 707, 236, and 236 nodules, respectively. The proportions of invasive lesions were 78.5, 78.8, and 80.9% in the three subsets, respectively. Most demographic, clinical, and radiological characteristics were broadly comparable across the three subsets. A modest difference was observed in vascular/bronchial convergence, which was present in 43.0% of the training subset, 38.1% of the validation subset, and 33.9% of the held-out test subset (*p* = 0.037).

**Table 2:** Clinical and radiological characteristics of the overall cohort and the training, validation, and held-out test subsets in one representative iteration of five-fold cross-validation.

Characteristic	Category	Overall, *n* = 1,179	Training set, *n* = 707	Validation set, *n* = 236	Held-out test set, *n* = 236	*p* value
Pathological invasiveness	Invasive lesions (MIA/IAC)	932 (79.1)	555 (78.5)	186 (78.8)	191 (80.9)	0.726
Age, years	—	49.82 ± 13.05	49.35 ± 13.10	50.41 ± 12.75	50.64 ± 13.21	0.315
Nodule diameter, mm	—	10.94 ± 5.95	10.80 ± 5.91	11.04 ± 6.09	11.28 ± 5.96	0.533
CYFRA21-1	—	1.36 (0.99–1.90)	1.37 (0.98–1.87)	1.33 (0.91–1.88)	1.37 (1.04–1.95)	0.628
SCC	—	0.70 (0.50–0.90)	0.70 (0.60–0.90)	0.70 (0.50–0.90)	0.80 (0.60–1.00)	0.106
CEA	—	1.40 (1.00–2.10)	1.40 (1.00–2.10)	1.40 (1.00–2.20)	1.45 (1.00–2.12)	0.967
NSE	—	11.12 (9.06–13.00)	11.34 (9.26–13.05)	10.91 (9.14–13.02)	10.80 (8.79–12.90)	0.596
Sex	Male	412 (34.9)	262 (37.1)	83 (35.2)	67 (28.4)	0.054
Nodule location	Left upper lobe	261 (22.1)	155 (21.9)	47 (19.9)	59 (25.0)	0.427
	Left lower lobe	221 (18.7)	135 (19.1)	38 (16.1)	48 (20.3)	
	Right upper lobe	367 (31.1)	220 (31.1)	76 (32.2)	71 (30.1)	
	Right middle lobe	101 (8.6)	67 (9.5)	18 (7.6)	16 (6.8)	
	Right lower lobe	229 (19.4)	130 (18.4)	57 (24.2)	42 (17.8)	
Underlying pulmonary disease	Yes	26 (2.2)	15 (2.1)	8 (3.4)	3 (1.3)	0.285
Family history of malignancy	Yes	82 (7.0)	51 (7.2)	13 (5.5)	18 (7.6)	0.606
History of extrathoracic malignancy	Yes	79 (6.7)	49 (6.9)	17 (7.2)	13 (5.5)	0.708
Smoking history	Yes	121 (10.3)	80 (11.3)	24 (10.2)	17 (7.2)	0.197
Density composition	Solid nodule (SN)	228 (19.3)	128 (18.1)	49 (20.8)	51 (21.6)	0.519
	Pure ground-glass nodule (pGGN)	425 (36.0)	267 (37.8)	77 (32.6)	81 (34.3)	
	Mixed ground-glass nodule (mGGN)	526 (44.6)	312 (44.1)	110 (46.6)	104 (44.1)	
Long spiculation	Yes	65 (5.5)	43 (6.1)	7 (3.0)	15 (6.4)	0.157
Short spiculation	Yes	152 (12.9)	87 (12.3)	25 (10.6)	40 (16.9)	0.091
Vacuole sign	Yes	222 (18.8)	125 (17.7)	52 (22.0)	45 (19.1)	0.332
Lobulation sign	Yes	194 (16.5)	109 (15.4)	47 (19.9)	38 (16.1)	0.268
Vascular/bronchial convergence	Yes	474 (40.2)	304 (43.0)	90 (38.1)	80 (33.9)	0.037
Pleural indentation	Yes	208 (17.6)	121 (17.1)	41 (17.4)	46 (19.5)	0.704
Shape	Irregular	91 (7.7)	51 (7.2)	19 (8.1)	21 (8.9)	0.687
Margin	Ill-defined	215 (18.2)	133 (18.8)	41 (17.4)	41 (17.4)	0.822

Data are presented as n (%) unless otherwise indicated. For binary characteristics, only the positive category is presented; for sex, only the male category is presented. Age and nodule diameter are presented as mean ± standard deviation, whereas tumour markers are presented as median (interquartile range). Percentages were calculated using available observations. *p* values compare the training, validation, and held-out test subsets and were calculated using one-way analysis of variance for age and nodule diameter, the Kruskal–Wallis test for tumour markers, and the chi-square test or Fisher’s exact test for categorical variables, as appropriate. AAH, atypical adenomatous hyperplasia; AIS, adenocarcinoma in situ; MIA, minimally invasive adenocarcinoma; IAC, invasive adenocarcinoma; SN, solid nodule; pGGN, pure ground-glass nodule; mGGN, mixed ground-glass nodule; SD, standard deviation.

Direct comparisons according to pathological invasiveness are presented in Table 3. Compared with glandular precursor lesions, invasive lesions occurred in older patients, had larger nodule diameters, and showed higher CYFRA21-1 concentrations. Smoking history was also more frequent among patients with invasive lesions. The overall distribution of nodule density differed between the two pathological groups: solid nodules and mixed ground-glass nodules were more frequent among invasive lesions, whereas pure ground-glass nodules were more frequent among glandular precursor lesions. Long and short spiculation, vacuole sign, lobulation, vascular/bronchial convergence, pleural indentation, irregular shape, and ill-defined margins were also more frequent among invasive lesions. Detailed estimates and *p* values are provided in Table 3.

**Table 3:** Clinical and radiological characteristics according to pathological invasiveness.

Characteristic	Category	Glandular precursor lesions (AAH/AIS), *n* = 247	Invasive lesions (MIA/IAC), *n* = 932	*p* value
Age, years	—	45.90 ± 11.27	50.86 ± 13.30	<0.001
Nodule diameter, mm	—	7.00 (5.00–8.00)	10.00 (7.00–15.00)	<0.001
CYFRA21-1	—	1.27 (0.95–1.63)	1.40 (1.00–1.94)	0.014
SCC	—	0.70 (0.57–0.90)	0.70 (0.50–0.90)	0.877
CEA	—	1.40 (1.00–2.10)	1.50 (1.00–2.18)	0.520
NSE	—	11.12 (9.16–12.80)	11.12 (9.06–13.00)	0.802
Sex	Male	73 (29.6)	339 (36.4)	0.054
Nodule location	Left upper lobe	60 (24.3)	201 (21.6)	0.699
	Left lower lobe	43 (17.4)	178 (19.1)	
	Right upper lobe	70 (28.3)	297 (31.9)	
	Right middle lobe	22 (8.9)	79 (8.5)	
	Right lower lobe	52 (21.1)	177 (19.0)	
Underlying pulmonary disease	Yes	6 (2.4)	20 (2.1)	0.979
Family history of malignancy	Yes	17 (6.9)	65 (7.0)	1.000
History of extrathoracic malignancy	Yes	15 (6.1)	64 (6.9)	0.764
Smoking history	Yes	10 (4.0)	111 (11.9)	<0.001
Density composition	Solid nodule (SN)	18 (7.3)	210 (22.5)	<0.001
	Pure ground-glass nodule (pGGN)	135 (54.7)	290 (31.1)	
	Mixed ground-glass nodule (mGGN)	94 (38.1)	432 (46.4)	
Long spiculation	Yes	4 (1.6)	61 (6.5)	0.004
Short spiculation	Yes	5 (2.0)	147 (15.8)	<0.001
Vacuole sign	Yes	10 (4.0)	212 (22.7)	<0.001
Lobulation sign	Yes	7 (2.8)	187 (20.1)	<0.001
Vascular/bronchial convergence	Yes	30 (12.1)	444 (47.6)	<0.001
Pleural indentation	Yes	4 (1.6)	204 (21.9)	<0.001
Shape	Irregular	8 (3.2)	83 (8.9)	0.005
Margin	Ill-defined	20 (8.1)	195 (20.9)	<0.001

Data are presented as n (%) unless otherwise indicated. For binary characteristics, only the positive category is presented; for sex, only the male category is presented. Age is presented as mean ± standard deviation. Nodule diameter and tumour-marker concentrations are presented as median (interquartile range). Percentages and statistical tests were based on available observations. *p* values were calculated using Welch’s t-test for age, the Wilcoxon rank-sum test for nodule diameter and tumour markers, and the chi-square test or Fisher’s exact test for categorical variables, as appropriate. Yates’ continuity correction was applied to 2 × 2 chi-square tests. AAH, atypical adenomatous hyperplasia; AIS, adenocarcinoma in situ; MIA, minimally invasive adenocarcinoma; IAC, invasive adenocarcinoma; SN, solid nodule; pGGN, pure ground-glass nodule; mGGN, mixed ground-glass nodule; SD, standard deviation.

### 3.2 Comparison of different image feature extractors

Five feature-extraction architectures were evaluated, including VGG-16, ResNet-50, DenseNet-121, ViT, and DINOv3. Their complete performance metrics are presented in Table 1.

DINOv3 achieved the highest point-estimate AUC of 0.729 (95% CI, 0.659–0.799), followed by DenseNet-121, ResNet-50, ViT, and VGG-16, with AUCs of 0.719, 0.718, 0.715, and 0.679, respectively. DINOv3 achieved an accuracy of 0.648, a sensitivity of 0.612, a specificity of 0.792, and an F1 score of 0.735. Pairwise DeLong tests based on the pooled out-of-fold predictions showed that the AUC of DINOv3 did not differ significantly from those of DenseNet-121, ResNet-50, VGG-16, or ViT (*p* = 0.129, 1.000, 1.000, and 0.320, respectively; Supplementary Table S3A). DINOv3 was therefore selected as the image feature extractor for the final framework based on its highest point-estimate AUC rather than statistically significant superiority over the alternative architectures. Qualitative PCA-based visualizations of the DINOv3-derived feature representations for representative pulmonary adenocarcinoma-spectrum lesions are presented in Figure 3. These visualizations were used solely to illustrate the spatial distribution of the extracted representations and were not included in quantitative model evaluation.

**Figure 3:**
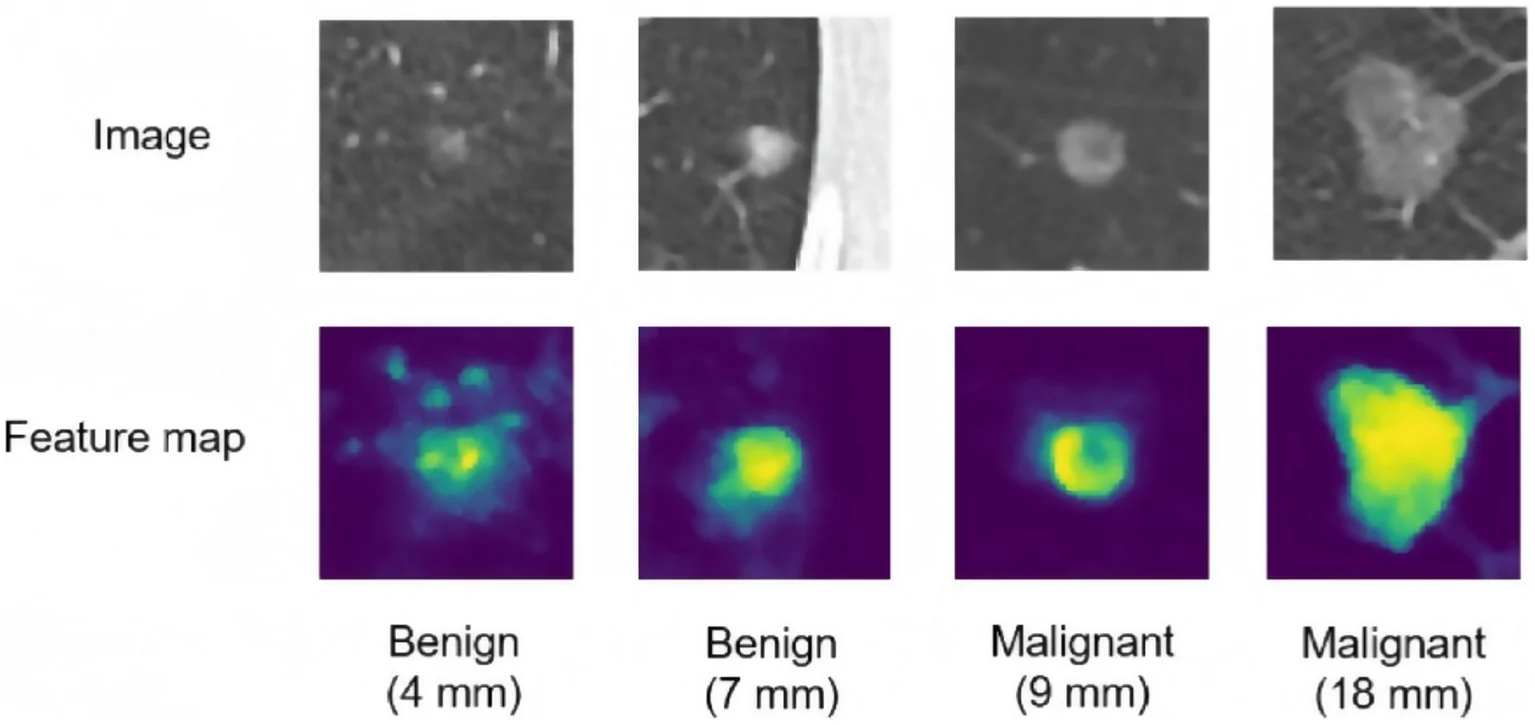
Representative CT images and corresponding DINOv3-derived feature maps of pulmonary nodules. The upper row shows the original CT images, and the lower row shows the corresponding qualitative feature visualizations generated from DINOv3-derived image representations. These visualizations were used only for qualitative interpretation and were not included in model training, model selection, or quantitative performance evaluation.

### 3.3 Comparison of different classification algorithms

Six classification algorithms were evaluated using the same extracted imaging and clinical representations: a fully connected neural classifier, logistic regression, support vector machine, random forest, XGBoost, and NCART. Their complete performance metrics are presented in Supplementary Table S1.

NCART achieved the highest point-estimate AUC of 0.847, followed by XGBoost, the fully connected neural classifier, random forest, logistic regression, and support vector machine, with AUCs of 0.843, 0.841, 0.839, 0.833, and 0.810, respectively. The DeLong test showed that NCART had a significantly higher AUC than SVM (*p* = 0.026). Its AUC did not differ significantly from those of the fully connected neural classifier, logistic regression, random forest, or XGBoost (*p* = 0.653, 0.244, 0.549, and 0.770, respectively; Supplementary Table S3B). NCART was selected as the classifier for the final framework on the basis of its highest point-estimate AUC.

### 3.4 Comparison of multimodal fusion strategies

To investigate the contribution of multimodal integration, we compared single-modality models, late fusion, and the proposed intermediate-fusion strategy.

The performance of the Gould score, clinical-data-only model, imaging-only model, late-fusion model, and intermediate-fusion model was compared, as presented in Supplementary Table S2. The intermediate-fusion model achieved the highest point-estimate AUC of 0.847, compared with 0.805 for late fusion, 0.742 for the clinical-data-only model, 0.736 for the Gould score, and 0.729 for the imaging-only model.

Pairwise DeLong tests showed that the intermediate-fusion model achieved significantly higher AUCs than the Gould score (*p* = 0.0018) and the clinical-data-only model (*p* = 0.0061). Its differences from the imaging-only model and the late-fusion model did not reach statistical significance (*p* = 1.000 and *p* = 0.075, respectively; Supplementary Table S3C).

The final DINOv3-based intermediate-fusion model with the NCART classifier was evaluated using the five-fold train–validation–test procedure described above. The held-out test-fold AUCs were 0.848, 0.864, 0.867, 0.882, and 0.822, respectively, with a mean AUC of 0.8566 and a range of 0.822–0.882 (Figure 4). No separate fixed test cohort was used outside the five-fold cross-validation framework.

**Figure 4:**
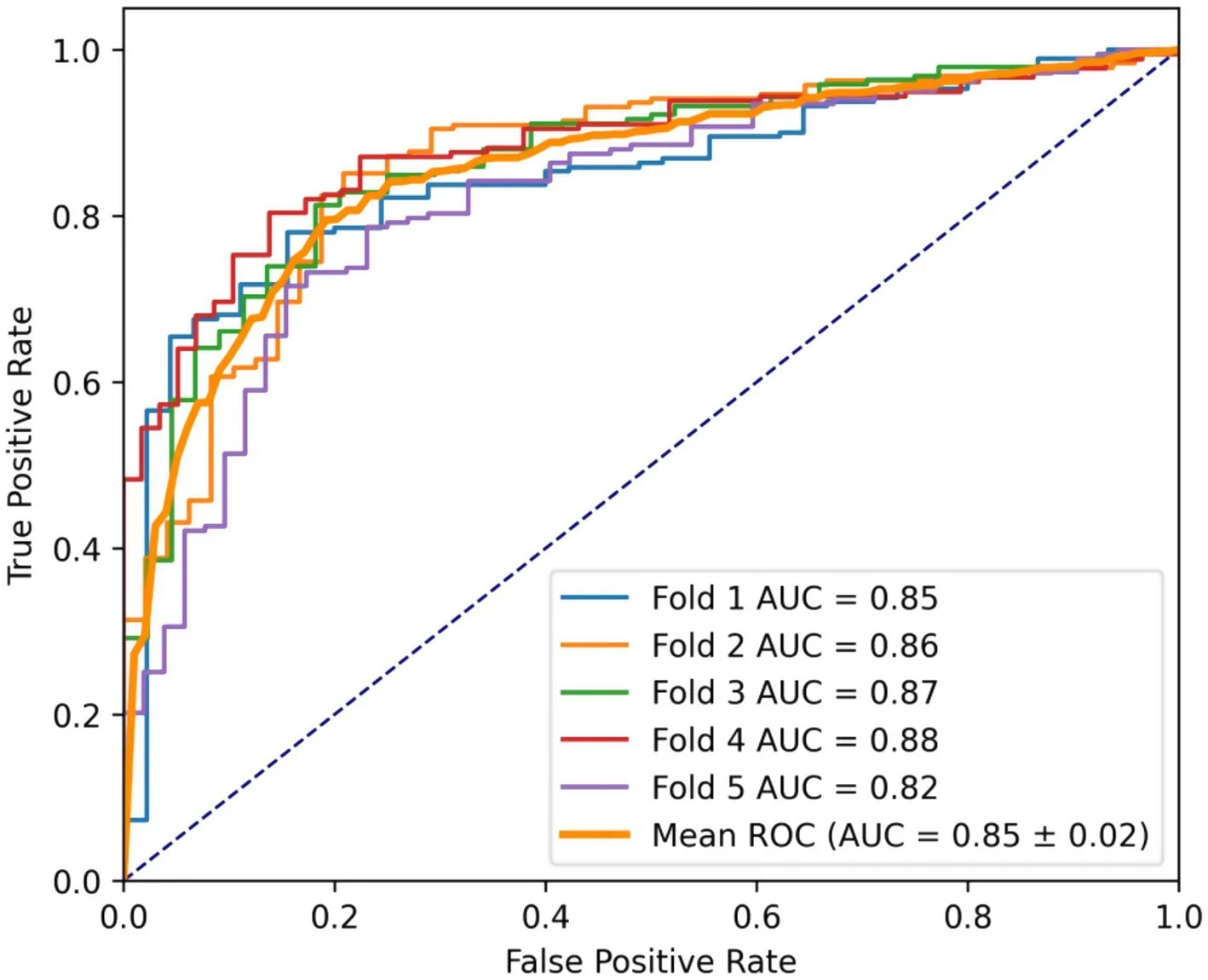
Five-fold cross-validated receiver operating characteristic curves of the final multimodal model for predicting pulmonary nodule invasiveness. The final model comprised the frozen DINOv3 feature extractor, intermediate multimodal fusion module, and NCART classifier. The held-out test-fold AUCs were 0.848, 0.864, 0.867, 0.882, and 0.822, respectively, yielding a mean AUC of 0.8566.

### 3.5 Prespecified secondary analysis distinguishing AIS from AAH

In the prespecified secondary subgroup analysis restricted to all pathologically confirmed AAH and AIS lesions, AIS was defined as the positive class and AAH as the negative class. The model achieved an AUC of 0.780 (95% CI, 0.549–0.999), with a sensitivity of 0.804 (95% CI, 0.668–0.893) and a specificity of 0.750 (95% CI, 0.301–0.954). Given the limited number of AAH and AIS lesions and the wide confidence intervals, these findings should be interpreted as exploratory.

## 4 Discussion

In this study, we developed and internally validated a multimodal artificial intelligence framework for distinguishing invasive pulmonary adenocarcinoma-spectrum lesions comprising MIA and IAC from glandular precursor lesions comprising AAH and AIS. The framework integrated high-dimensional CT representations extracted using a frozen DINOv3 backbone with clinical variables through an attention-based intermediate-fusion strategy, followed by classification using NCART. Three principal findings emerged. First, DINOv3 and NCART achieved the highest point-estimate AUCs among the evaluated feature extractors and classifiers, respectively, although most pairwise differences did not reach statistical significance. Second, the intermediate-fusion model achieved significantly higher AUCs than the Gould score and the clinical-data-only model, but not the imaging-only or late-fusion models. Third, a prespecified secondary analysis restricted to pathologically confirmed AAH and AIS lesions suggested potential discrimination between AIS and AAH, although the wide confidence intervals require cautious interpretation.

Conventional CT morphology remains important in the assessment of pulmonary nodule invasiveness. Nodule size, density, the proportion of solid components, lobulation, and spiculation are established radiological characteristics considered during pulmonary nodule evaluation (Wang et al., 2024; MacMahon et al., 2017; Chen et al., 2023). Previous studies have also associated increasing nodule diameter and a greater solid component with a higher likelihood of pathological invasiveness (Kakinuma et al., 2015). Nevertheless, conventional morphological assessment is partly subjective, may show limited reproducibility, and may be insufficient for reliably differentiating pGGNs from mGGNs (Raad et al., 2025). Consistent with this clinical context, the pathological-group comparison in the present study showed that invasive lesions were larger and more frequently exhibited solid or mixed ground-glass density, spiculation, vacuole sign, lobulation, vascular or bronchial convergence, pleural indentation, irregular shape, and ill-defined margins. Conversely, pure ground-glass nodules were more frequent among AAH and AIS lesions. These comparisons provide clinically interpretable context for the prediction task, but the conventional radiological characteristics were not manually selected as independent imaging inputs to the DINOv3 branch. Instead, the image branch learned high-dimensional representations directly from CT image patches.

Radiomics converts medical images into quantitative data and can capture textural, morphological, and higher-order imaging information that may not be readily appreciable through visual assessment alone (Chen et al., 2023; Vernuccio et al., 2020; Lambin et al., 2017). In the present comparison, DINOv3 achieved the highest point-estimate AUC among the evaluated feature extractors. Its large-scale self-supervised pretraining may have provided transferable visual representations before application to pulmonary CT images, potentially enabling the model to encode structural and contextual patterns related to nodule morphology, internal density distribution, and the surrounding pulmonary environment. This strategy may be particularly relevant in medical-imaging studies with limited pathologically labeled samples.

However, the present findings do not establish the intrinsic superiority of DINOv3 over the alternative architectures. DINOv3 used officially released pretrained weights and remained frozen throughout model development, whereas DenseNet-121, ResNet-50, VGG-16, and ViT were trained from random initialization. The comparison therefore evaluated the implemented training strategies rather than isolating the independent effect of architecture. Moreover, the pairwise DeLong comparisons between DINOv3 and the alternative feature extractors did not reach statistical significance. The results should consequently be interpreted as supporting the feasibility of using frozen DINOv3 representations for pulmonary CT analysis rather than demonstrating unconditional architectural superiority.

Pulmonary nodules are three-dimensional structures, and recent methods have applied three-dimensional convolutional networks and continuous shape-embedding approaches to model volumetric morphology, internal heterogeneity, and nodule boundaries (Gu et al., 2025). In contrast, the present framework primarily analysed two-dimensional CT image patches. This design reduced computational complexity and facilitated the application of a pretrained visual foundation model, but it may not fully capture volumetric growth patterns, complex internal structures, or spatial relationships across adjacent CT sections. Future studies should directly compare two-dimensional and three-dimensional implementations under otherwise equivalent data-partitioning and validation conditions.

The downstream classifier also influenced the point estimates of model performance. NCART achieved the highest point-estimate AUC among the six evaluated classifiers and significantly outperformed the support vector machine. However, its differences from the fully connected neural classifier, logistic regression, random forest, and XGBoost were not statistically significant. NCART may provide flexible nonlinear partitioning of fused imaging and clinical representations through its tree-based structure, but the current results do not demonstrate that this structural property is consistently superior to other conventional or nonlinear classifiers. Its inclusion in the final framework was therefore based on the highest observed point-estimate AUC under the prespecified comparison rather than broad statistical superiority. Further evaluation in larger independent datasets is required to determine whether this point-estimate advantage is reproducible.

Pathological examination remains the reference standard for determining pulmonary nodule subtype. However, obtaining tissue generally requires biopsy or surgical resection and may be associated with procedure-related complications, including pneumothorax (Kim et al., 2023; Kang, 2023). A non-invasive framework that estimates pathological invasiveness using routinely available CT images and clinical information may therefore provide complementary information before definitive pathological diagnosis. Nevertheless, such a model should be regarded as an investigational decision-support approach rather than a replacement for pathological examination or clinical judgment.

Relying exclusively on image-derived representations may overlook relevant clinical context, whereas clinical variables alone may not adequately capture the complex morphological and spatial information contained in CT images. Current pulmonary nodule management is based on the combined consideration of imaging characteristics, clinical risk factors, and physician assessment, and the Fleischner Society recommendations also support comprehensive evaluation rather than reliance on a single characteristic (MacMahon et al., 2017). In the present study, the intermediate-fusion model achieved significantly higher AUCs than the Gould score and the clinical-data-only model, suggesting that CT-derived representations provided discriminative information beyond the evaluated clinical variables alone.

The bidirectional cross-attention mechanism used in the intermediate-fusion framework may permit clinical and imaging representations to interact before final classification, thereby allowing context-dependent weighting of information from the two modalities. However, the intermediate-fusion model did not significantly outperform either the imaging-only model or the late-fusion model. The current findings therefore support the feasibility of multimodal intermediate fusion and indicate added discrimination relative to clinical assessment alone, but they do not establish its definitive superiority over imaging-based prediction or alternative fusion strategies.

The primary endpoint of this study was the binary distinction between invasive lesions comprising MIA and IAC and glandular precursor lesions comprising AAH and AIS. This grouping was selected to address the principal clinical question of pathological invasiveness and was not intended to represent simultaneous four-class classification. The AAH-versus-AIS analysis was a separate prespecified subgroup analysis restricted to all pathologically confirmed AAH and AIS lesions. It was not a sequential second stage triggered by the predictions generated by the primary model.

In this secondary analysis, AIS was defined as the positive class and AAH as the negative class. The observed AUC of 0.780 suggested potential discrimination between these two precursor categories. However, the limited number of AAH and AIS lesions resulted in wide confidence intervals for the performance estimates. The secondary finding should therefore be considered exploratory and requires confirmation in substantially larger independent cohorts enriched for precursor lesions.

Management of indolent pulmonary nodules requires balancing timely identification of clinically relevant disease against the risks of overdiagnosis, overtreatment, and unnecessary invasive procedures. Previous studies have investigated individualized imaging follow-up and management strategies in this setting (Jiang et al., 2026; Wang et al., 2025; Liang et al., 2025; Ye et al., 2025). However, the present retrospective study did not evaluate whether use of the proposed model could improve treatment selection, safely alter CT surveillance intervals, reduce unnecessary procedures, or improve patient outcomes. The current findings therefore do not support specific recommendations regarding surgical intervention or follow-up schedules.

The present study also did not directly compare the proposed framework with established pulmonary nodule risk-assessment or management systems, including PKU-LCSMS, C-Lung-RADS, and PulmoSeek Plus. These approaches may partly address different clinical questions, and no conclusion regarding comparative superiority can be drawn from the current results. The findings suggest only that foundation-model-based multimodal learning may provide complementary information for distinguishing glandular precursor lesions from invasive lesions. Direct head-to-head comparisons, external multicentre validation, and prospective clinical-impact studies are required before the clinical role of the proposed framework can be determined.

Several limitations should be acknowledged. First, this was a retrospective single-centre study. Although rotating held-out test folds provided internal validation, the transportability and generalisability of the model remain unknown, and independent multicentre and prospective validation is required. Second, the pathological classes were imbalanced, and the numbers of AAH and AIS lesions were particularly limited, resulting in substantial uncertainty in the secondary subgroup analysis. Larger datasets with more balanced pathological categories are required to obtain more precise performance estimates.

Third, DINOv3 was used as a frozen feature extractor pretrained on natural images, whereas the CNN-based models and ViT were trained from random initialization. This design allowed comparison of the implemented modeling strategies but did not constitute an architecture-controlled experiment. Future studies could assess domain-specific adaptation, medical image-oriented pretraining, or equivalent pretraining conditions across candidate architectures. Fourth, the imaging analysis was primarily based on non-contrast two-dimensional CT inputs and did not explicitly model full three-dimensional nodule geometry, longitudinal changes, contrast-enhanced imaging, genomic characteristics, or metabolomic information.

Finally, this study evaluated retrospective discrimination rather than external calibration, integration into clinical workflow, changes in physician decision-making, or patient-level outcomes. The applicability of the framework to pulmonary nodules outside the adenocarcinoma spectrum, including squamous cell carcinoma and small-cell lung cancer, also remains uncertain. These limitations should be addressed before the proposed framework is considered for clinical implementation.

## 5 Conclusion

In summary, this study developed an internally validated multimodal framework that integrated CT representations extracted using a frozen DINOv3 backbone with clinical variables through attention-based intermediate fusion and NCART classification. Across five rotating held-out test folds, the final model achieved a mean AUC of 0.8566 for distinguishing invasive lesions comprising MIA and IAC from glandular precursor lesions comprising AAH and AIS.

DINOv3 and NCART were selected on the basis of the highest point-estimate AUCs among the evaluated feature extractors and classifiers, respectively, although most comparisons with alternative approaches did not reach statistical significance. The intermediate-fusion model showed statistically significant improvements over the Gould score and the clinical-data-only model, but not over the imaging-only or late-fusion models. A prespecified secondary analysis suggested potential discrimination between AIS and AAH, although the limited number of precursor lesions requires cautious interpretation. External multicentre and prospective validation is required before clinical implementation.

## Data Availability

The raw data supporting the conclusions of this article will be made available by the authors, without undue reservation.
