# Supplementary material for: Imaging-based development and validation of artificial intelligence models for lung adenocarcinoma precursor lesions and early lung adenocarcinoma presenting as pulmonary nodules
Source: Front Artif Intell. 2026 Sep 15;9:1889248. doi: 10.3389/frai.2026.1889248 (PMC13619932; doi:10.3389/frai.2026.1889248)
Supplement: Supplementary file 1 [file Data_Sheet_1.DOCX]

**Supplementary Material**

**Supplementary Figure S1. Architectures of the imaging-only and clinical-data-only baseline models.**
The upper architecture shows the imaging-only model. CT representations were extracted using the pretrained and frozen DINOv3 backbone, processed by the trainable Attention Probe with positional encoding, and subsequently passed to the classifier. The lower architecture shows the clinical-data-only model, in which encoded categorical and continuous clinical features were concatenated and passed directly to the classifier. The flame symbol indicates trainable modules, the snowflake symbol indicates the frozen DINOv3 backbone, the sphere symbol indicates positional encoding, C indicates concatenation, and cls indicates the classification token.

**Supplementary Figure S2. Architecture of the multimodal late-fusion baseline model.**

CT representations were extracted using the pretrained and frozen DINOv3 backbone and processed by the trainable Attention Probe with positional encoding. Categorical and continuous clinical features were encoded and concatenated to form the clinical representation. The imaging classification token and the clinical representation were processed separately and concatenated immediately before the final classifier. The flame symbol indicates trainable modules, the snowflake symbol indicates the frozen DINOv3 backbone, the sphere symbol indicates positional encoding, C indicates concatenation, and cls indicates the classification token.

**Supplementary Table 1: Performance Comparison of Different Classifiers**

|  | **AUC(95% CI)** | **ACC(95% CI)** | **PREC(95% CI)** | **SEN(95% CI)** | **SPEC(95% CI)** | **F1(95% CI)** |
| --- | --- | --- | --- | --- | --- | --- |
| FC | 0.841  (0.783-0.899) | 0.758  (0.700-0.809) | 0.972  (0.930-0.989) | 0.723  (0.655-.781) | 0.911  (0.793-0.965) | 0.829  (0.784-0.869) |
| LR | 0.833  (0.772-0.894) | 0.750  (0.691-0.801) | 0.971  (0.929-0.989) | 0.712  (0.644-0.772) | 0.911  (0.793-0.965) | 0.822  (0.773-0.863) |
| SVM | 0.810  (0.741-0.879) | 0.767  (0.709-0.816) | 0.947  (0.900-0.973) | 0.754  (0.688-0.810) | 0.822  (0.687-0.907) | 0.840  (0.795-0.880) |
| RF | 0.839  (0.778-0.889) | 0.814  (0.759-0.858) | 0.940  (0.893-0.967) | 0.822  (0.762-0.870) | 0.778  (0.637-0.875) | 0.877  (0.837-0.914) |
| XGB | 0.843  (0.786-0.900) | 0.814  (0.759-0.858) | 0.951  (0.906-0.975) | 0.812  (0.750-0.861) | 0.822  (0.687-0.907) | 0.876  (0.837-0.913) |
| NCART | 0.847  (0.789-0.906) | 0.809  (0.754-0.854) | 0.951  (0.906-0.975) | 0.806  (0.744-0.856) | 0.822  (0.687-0.907) | 0.873  (0.833-0.909) |

**Supplementary Table S2: Performance Comparison of Different Modalities and Fusion Strategies**

|  | **AUC(95% CI)** | **ACC(95% CI)** | **PREC(95% CI)** | **SEN(95% CI)** | **SPEC(95% CI)** | **F1(95% CI)** |
| --- | --- | --- | --- | --- | --- | --- |
| Gould | 0.736  (0.669-0.804) | 0.657  (0.594-0.714) | 0.974  (0.927-0.991) | 0.592  (0.521-0.659) | 0.933  (0.821-0.977) | 0.736  (0.677-0.788) |
| Clinical data | 0.742  (0.674-0.809) | 0.627  (0.564-0.686) | 0.956  (0.901-0.981) | 0.565  (0.495-0.634) | 0.889  (0.765-0.952) | 0.711  (0.648-0.768) |
| Images | 0.729  (0.659-0.799) | 0.648  (0.585-0.706) | 0.920  (0.859-0.956) | 0.612  (0.540-0.678) | 0.792  (0.657-0.883) | 0.735  (0.682-0.788) |
| Late fusion | 0.805  (0.743-0.867) | 0.691  (0.629-0.746) | 0.961  (0.912-0.983) | 0.644  (0.574-0.708) | 0.889  (0.765-0.952) | 0.771  (0.716-0.820) |
| Intermediate fusion | 0.847  (0.789-0.906) | 0.809  (0.754-0.854) | 0.951  (0.906-0.975) | 0.806  (0.744-0.856) | 0.822  (0.687-0.907) | 0.873  (0.833-0.909) |

**Supplementary Table 3. Pairwise DeLong P-value matrices for feature extractors, classifiers, and modality/fusion strategies based on pooled out-of-fold predictions**

**Supplementary Table 3A. Pairwise DeLong P values among feature extractors**

|  | **DenseNet-121** | **ResNet-50** | **VGG-16** | **ViT** | **DINOv3** |
| --- | --- | --- | --- | --- | --- |
| DenseNet-121 | 1.000 | 0.241 | 0.004 | 0.422 | 0.129 |
| ResNet-50 | 0.241 | 1.000 | 0.342 | 1.000 | 1.000 |
| VGG-16 | 0.004 | 0.342 | 1.000 | 1.000 | 1.000 |
| ViT | 0.422 | 1.000 | 1.000 | 1.000 | 0.320 |
| DINOv3 | 0.129 | 1.000 | 1.000 | 0.320 | 1.000 |

**Supplementary Table 3B. Pairwise DeLong P values among classifiers**

|  | **FC** | **LR** | **SVM** | **RF** | **XGBoost** | **NCART** |
| --- | --- | --- | --- | --- | --- | --- |
| FC | 1.000 | 0.260 | 0.089 | 0.949 | 0.912 | 0.653 |
| LR | 0.260 | 1.000 | 0.175 | 0.523 | 0.523 | 0.244 |
| SVM | 0.089 | 0.175 | 1.000 | 0.042 | 0.056 | 0.026 |
| RF | 0.949 | 0.523 | 0.042 | 1.000 | 0.845 | 0.549 |
| XGBoost | 0.912 | 0.523 | 0.056 | 0.845 | 1.000 | 0.770 |
| NCART | 0.653 | 0.244 | 0.026 | 0.549 | 0.770 | 1.000 |

**Supplementary Table 3C. Pairwise DeLong P values among modality and fusion strategies**

|  | **Gould** | **Clinical data** | **Images** | **Late fusion** | **Intermediate fusion** |
| --- | --- | --- | --- | --- | --- |
| Gould | 1.000 | 0.896 | 1.000 | 0.066 | 0.002 |
| Clinical data | 0.896 | 1.000 | 1.000 | 0.103 | 0.006 |
| Images | 1.000 | 1.000 | 1.000 | 1.000 | 1.000 |
| Late fusion | 0.066 | 0.103 | 1.000 | 1.000 | 0.075 |
| Intermediate fusion | 0.002 | 0.006 | 1.000 | 0.075 | 1.000 |

Abbreviations: AUC, area under the receiver operating characteristic curve; ΔAUC, difference in AUC calculated as the AUC of model 1 minus that of model 2; FC, fully connected neural classifier; LR, logistic regression; SVM, support vector machine; RF, random forest; XGBoost, extreme gradient boosting; ViT, Vision Transformer.

Pairwise comparisons of correlated AUCs were conducted using the DeLong test. All P values were two-sided, and P<0.05 was considered statistically significant.
